# Hypoglycemic and H_2_O_2_-induced oxidative injury protective effects and the phytochemical profiles of the ethyl acetate fraction from *Radix Paeoniae Alba*

**DOI:** 10.3389/fnut.2023.1126359

**Published:** 2023-02-24

**Authors:** Lu Zhang, Chun-yan Peng, Pei-xin Wang, Linju Xu, Jia-hui Liu, Xing Xie, Ling Lu, Zong-cai Tu

**Affiliations:** ^1^National R&D Center of Freshwater Fish Processing, College of Life Science, Jiangxi Normal University, Nanchang, Jiangxi, China; ^2^Jiangxi Deshang Pharmaceutical Co., Ltd., Yichun, Jiangxi, China; ^3^State Key Laboratory of Food Science and Technology, Nanchang University, Nanchang, Jiangxi, China

**Keywords:** *Radix Paeoniae Alba*, hypoglycemic activity, polyphenols, HPLC-QTOF-MS/MS, oxidative stress

## Abstract

*Radix Paeonia Alba* (RPA) is often used as food and medicine. This study aimed to enrich and identify the antioxidant and hypoglycemic bioactive compounds from RPA. The results indicated that the ethyl acetate fraction (EAF) showed the highest total phenolic content, DPPH, ABTS^+^ scavenging ability, and α-glucosidase inhibition ability (IC_50_ = 7.27 μg/ml). The EAF could alleviate H_2_O_2_-induced oxidative stress in HepG2 cells by decreasing the MDA and ROS levels, improving cell apoptosis, increasing the enzyme activity of GPX-Px, CAT, SOD, Na^+^/K^+^-ATP, and Ca^2+^/Mg^2+^-ATP, and stimulating T-AOC expression, which also enhanced the glucose uptake of insulin-resistant HepG2 cells. In addition, the EAF significantly reduced the fasting blood glucose level and improved glucose tolerance in diabetic mice. An HPLC-QTOF-MS/MS analysis displayed that procyanidin, digallic acid isomer, methyl gallate, tetragalloylglucose isomer, dimethyl gallic acid, and paeoniflorin were the major compounds in the EAF. These findings are meaningful for the application of the EAF in the medicinal or food industry to prevent and treat oxidative stress and diabetes mellitus.

## 1. Introduction

Diabetes mellitus (DM) is a worldwide-prevalent chronic disease inherited from natural or acquired insufficiencies and ineffectiveness of insulin secretion. It can be further divided into type I diabetes, type II diabetes (T2D), gestational diabetes mellitus, and others. Over 90% of the cases of type I diabetes are T2D (1). The International Diabetes Federation estimated that 537 million adults are living with diabetes in 2021 and that the amount will increase by 74 million compared with 2019, indicating an increment of 16%, and it is expected to reach 783 million by 2045 (https://diabetesatlas.org/data/en/country/42/cn.html). T2D is a complex metabolic abnormality characterized by chronic hyperglycemia and impaired pancreatic β-cell function and may negatively influence the structure and function of many organ systems by increasing the risk of cardiovascular disease, heart failure, diabetic kidney disease, diabetic retinopathy, etc. (2–4). Currently, mitigating glucose absorption, promoting insulin secretion, alleviating insulin resistance, inhibiting glucagon-like peptide-1 (GLP-1) receptor, sodium-glucose cotransporter-2, and diet control are the predominant modalities of T2D management (5). Meanwhile, oxidative stress is an important piece for understanding the complex mechanism involved in the development of diabetes and its complications (6, 7).

Reactive oxygen species (ROS), including singlet oxygen (O_2_), superoxide anion radicals (O_2_), hydrogen peroxide (H_2_O_2_), and hydroxyl radicals (OH), are formed during the respiration process and play an important role in biological functions, such as cell proliferation, apoptosis, and signal transduction in organisms (8). While excessive ROS will cause impaired glycometabolism in the liver, enhance insulin resistance in the liver and skeletal muscle cells, and reduce the function of pancreatic β-cells, it also promotes the development of diabetes (7). Antioxidants can alleviate oxidative stress, prevent or delay ROS-triggered apoptosis, and might be a reasonable way to treat diabetes and other metabolic syndromes. Antioxidant enzymes, antioxidants, and proteins that separate transition metals are the main ROS defense systems in an organism. In the antioxidant enzyme protection system, superoxide dismutase (SOD), glutathione peroxidase (GSH-Px), and catalase (CAT) can help to scour free radicals and reduce or eliminate oxidative damage (9, 10). Antioxidants, natural substances obtained from natural plants and synthetic chemicals that show strong radical scavenging ability, can prevent oxidative injury by removing excessive ROS, decreasing malondialdehyde (MDA), and enhancing the activity of antioxidant enzymes (11). For example, N-acetylcysteine could improve insulin secretion and insulin signaling by mitigating oxidative stress (6). However, the application of synthetic antioxidants was limited due to their potential teratogenicity, carcinogenicity, and mutagenicity (12). Thus, compounds derived from foods or herbs that exhibit low toxicity and high antioxidant ability may reduce oxidative damage by balancing the ROS level in the body.

*Radix Paeonia Alba* (RPA) is the dry root of herbaceous peony and is widely used in Chinese food due to its rich nutrition and health care function, especially in various stews and soups, such as stewed RPA with pig's feet, stewed RPA with pigeon, paeonia–glycyrrhiza soup, oyster–RPA soup, and papaya–RPA soup. Modern pharmacological studies have shown that RPA can regulate the immune, digestive, and cardiovascular systems (13). Di et al. (14) found that paeoniflorin pretreatment drastically attenuates the ROS level in H_2_O_2_-induced Schwann cell injury. The total glucosides of paeonia clearly improved the kidney-related symptoms in diabetic rats (15). Meanwhile, several bodies of literature have reported that the crude extract or pure compounds of RPA scavenged radicals (DPPH, ABTS^+^, etc.), inhibited nitric oxide (NO) production, and prevented diabetes-associated renal damage (16). However, the *in vitro* and *in vivo* antioxidant and hypoglycemic abilities of an RPA extract and its main active compounds still need to be researched further.

In this study, RPA was extracted and fractionated with different solvents, and the total phenolic content (TPC), radical scavenging, and α-glucosidase inhibition activities were evaluated to screen the fraction with the strongest hypoglycemic and antioxidant activities. Then, HepG2 cell models with oxidative injury induced by H_2_O_2_ and insulin resistance were applied to investigate the effect of RPA and its fractions on oxidative stress and glucose absorption, respectively. The *in vivo* hypoglycemic and oral glucose tolerance test (OGTT) abilities were evaluated with db/db mice. Finally, the main chemical composition of the RPA fraction with the strongest activity was identified by high-performance liquid chromatography-tandem quadrupole time-of-flight mass spectrometry (HPLC-ESI-QTOF-MS/MS).

## 2. Materials and methods

### 2.1. Materials and chemicals

Dried *Radix Alba Paeoniae* was purchased from Anqing Chunyuan pharmacy in Anqing city, Anhui province, China on June 2021. Analytic-grade chloroform, ethyl acetate, and *n*-butanol and chromatographic-grade acetonitrile and formic acid were from Aladdin Reagent Int. (Shanghai, China). Metformin, acarbose, 1,1-diphenyl-2-picrylhydrazyl (DPPH), 2,2-azinobis-(3-ethylbenzothiazoline-6-sulfonic acid) (ABTS), α-glucosidase, and *p*-nitrophenyl-α-D-galactopyranoside (pNPG) were purchased from Sigma-Aldrich (St. Louis, MO, USA). Human hepatocellular carcinoma cells (HepG2) and culture media were purchased from the BeNa Culture Collection (Beijing, China). All other chemicals were of analytical grade and from Sinopharm Chemical Reagent Co., Ltd. (Shanghai, China).

### 2.2. Preparation of samples

Dried RPA was pulverized into powder by a disintegrator and soaked for 24 h in 70% ethanol solution (m/v, 1:20) at room temperature. After 7 days, the mixtures were filtered and the residues were extracted for two times under the same extraction conditions. The supernatants obtained through three times of extraction were combined and concentrated to yield the crude extract. Finally, the extracts were dissolved in distilled water and fractionated by chloroform, ethyl acetate, and *n*-butanol sequentially to yield the chloroform fraction (DCF), the ethyl acetate fraction (EAF), and the *n*-butanol fraction (*n*BuF) for further analyses.

### 2.3. Determination of the total phenolic content

The TPC was quantified by the Folin-Ciocalteau method (17) with some modifications. Briefly, 200 μl of properly diluted samples or standard were mixed with 100 μl of Folin-Ciocalteau reagent. After 5 min, 300 μl of Na_2_CO_3_ (7.5%, w/v) and 1.0 ml of water were added. After 30 min, the absorbance at 765 nm was measured using a microplate reader (Biotek, Vermont, USA). The TPC was calculated based on the calibration curve plotted using gallic acid (0–200 μg/ml) and the results were expressed as μg of gallic acid equivalents per gram of extract [(μg GAE)/g E].

### 2.4. Determination of DPPH·and ABTS^+^ scavenging ability

The DPPH·and ABTS^+^ scavenging activity were determined according to the methods reported in our previous study (2). Freshly prepared DPPH·or ABTS^+^ working solution (150 μl) was mixed with 50 μl of the sample at various concentrations in 96-well microplates. After 30 or 6 min of incubation at room temperature, the absorbance was measured at 517 or 734 nm. Ethanol and quercetin were used as the negative control and positive control, respectively. The DPPH·and ABTS^+^ scavenging activity were expressed as the IC_50_ value, which was calculated by a nonlinear curve fitting of percentage inhibition ratio vs. sample concentration (μg/ml) using Origin 2019 (OriginLab Co., US).

### 2.5. Evaluation of oxidative damage protection at the cellular level

#### 2.5.1. Cell cultivation and cell viability assay

HepG2 cells were cultured in Dulbecco's Modified Eagle's Medium (DMEM) supplemented with 10% fetal bovine serum (FBS), 100 U/ml penicillin, and 100 μg/ml streptomycin. The cells were grown at 37°C under a humidified 5% CO_2_ atmosphere. The CCK-8 method was used to determine cell viability (18). The HepG2 cells were seeded in 96-well plates at a density of 1.0 × 10^4^ cells/well and incubated for 24 h. Then, the cells were exposed to 10–300 μg/ml of the EAF or PBS for 24 h, and the cell viability was measured by a CCK-8 kit.

For oxidative damage protection analysis, the blank group, the control group, and the EAF group were set after 24 h of adherent incubation. The EAF and control groups were treated with 10–300 μg/ml of EAF and serum-free DMEM for 24 h, followed by the exposure of H_2_O_2_ for another 4 h. The blank group was treated with serum-free DMEM for 28 h. Finally, the cell viability was measured by a CCK-8 kit.

#### 2.5.2. Intracellular ROS level assay

The intracellular ROS level was determined using 2′7′-dichlorofluorescein diacetate (DCFH-DA) staining and flow cytometry (14). The HepG2 cells were seeded in 6-well plates (1 × 10^5^ cells/well), cultured in a humidified incubator with 5% CO_2_ at 37°C for 24 h, and treated with different concentrations of EAF and 300 μM of H_2_O_2_ in sequence. The cells were then stained with 10 μM of DCFH-DA in serum-free DMEM at 37°C for 30 min in darkness and washed two times with PBS and followed by digestion with trypsin and centrifugation at 1,000 rpm for 5 min. The fluorescent intensity was measured by an Accuri C6 flow cytometry (Becton, Dickinson and Company, USA) at an excitation wavelength of 488 nm, and the intracellular redox status was calculated based on the ratio of green vs. yellow.

#### 2.5.3. Measurement of MDA level and enzyme activity

HepG2 cells at the log phase were prepared as single-cell suspensions and seeded into 6-well plates (1 × 10^6^ cells/well) at 37°C for 24 h. After treatment with the EAF for 24 h, the cells were incubated with H_2_O_2_ for 4 h, washed two times with a PBS solution and lysed in lysis buffer (Biyuntian Biotechnology Co., Ltd, Shanghai, China). A BCA protein assay kit (Jiancheng Bioengineering Institute, Nanjing, China) was used to measure the intracellular protein content. The malondialdehyde (MDA) content, total antioxidant ability (T-AOC), catalase (CAT), glutathione peroxidase (GSH-Px), superoxide dismutase (SOD), Na^+^/K^+^-adenosine triphosphate (Na^+^/K^+^-ATP) and Ca^2+^/Mg^2+^-adenosine triphosphate (Ca^2+^/Mg^2+^-ATP) enzyme activity were determined with the corresponding assay kits according to the manufacturer's instructions (Jiancheng Bioengineering Institute, Nanjing, China).

#### 2.5.4. Detection of HepG2 apoptosis

Cell apoptosis was measured by AnnexinV FITC/PI staining and flow cytometry (14). HepG2 cells were cultured as described in Section 2.5.1. After digestion with trypsin containing EDTA (Solarbio, China), the cells were centrifuged at 1,000 *g* for 5 min at 4°C and washed two times with precooled PBS solution. Then, 1.0 × 10^6^ cells were collected and centrifuged at 1,000 *g* for 5 min and allowed to react with 5.0 μl of Annexin V-FITC at 4°C for 15 min in darkness, followed by incubation with 10 μl of PI staining solution. After incubation at 4°C for 5 min, the early, viable, late, and apoptotic cells were collected, and the percentage ratio was analyzed with an Accuri C6 flow cytometry. The x and y coordinates refer to the fluorescence intensities of annexin V and PI, respectively.

### 2.6. α-glucosidase inhibition assay

The inhibition activity of α*-*glucosidase was assessed by the previous method (18). Properly diluted samples (50 μl) were incubated with 50 μl of the α-glucosidase solution (0.2 U/ml) at 37°C for 10 min prior to the addition of 50 μl *p*NPG. After 50 min, 100 μl of the 0.2 M Na_2_CO_3_ solution was added to stop the reaction, absorbance was measured at 405 nm against a blank without α-glucosidase, and the system without sample was run in parallel as the control group. Acarbose was used as the positive control, and the IC_50_ value was used to evaluate the inhibition ability.

### 2.7. Determination of glucose consumption by IR-HepG2 cells

An insulin resistance (IR) model was induced as indicated in a previous study (3). HepG2 cells were seeded in 96-well plates at 2 × 10^5^ cells/well (100 μl/well) and cultured for 24 h. The cells were washed with PBS three times, treated with serum-free high-glucose DMEM (4.5 g/L) containing 1.0 mM insulin, and incubated at 37°C under 5% CO_2_ atmosphere for 36 h to induce the IR group. The cells cultured in high-glucose DMEM supplemented with 10% FBS and 1% antibiotic antimycotic solution were taken as the negative control group (Con). Then, the EAF solutions were added to the IR model cells for 24 h. The glucose content in the culture was detected using a glucose assay kit according to the manufacturer's instructions (Jiancheng Bioengineering Institute, Nanjing, China). The glucose consumption was calculated as follows: Glucose consumption = Cm–Cs, where Cm and Cs (mmol/L) refer to the glucose content in cells before and after 24 h of incubation, respectively.

### 2.8. *In vivo* hypoglycemic ability evaluation

#### 2.8.1. Animal experiments

A total of 508-week-old male C57BL6/J mice, including 40 db/db and 10 db/m mice, were purchased from Changzhou Cavens Laboratory Animal Co., Ltd [license key: SCXK (Su) 2016-0010, Jiangsu, China]. All experimental protocols in this research were approved by the Committee on the Ethics of Animal Experiments of the Jiangxi Normal University (Permission Number: JNU20210311-001). The breeding environment of mice was strictly controlled (25 ± 2°C, relative humidity 50 ± 5%, and 12/12 h diurnal cycle). After 1 week of adaptive feed, the 40 db/db mice were randomly divided into 5 groups: the diabetes group (Mod), the metformin group (250 mg/kg body weight per day, Met250), the low-dose group (100 mg/kg body weight per day, EAF100), the middle-dose group (250 mg/kg body weight per day, EAF250), and the high-dose group (400 mg/kg body weight per day, EAF400) and fed with D12451 during the period of drug administration. The non-diabetic mice (db/m) fed with D12450B only were used as the control group (Con). The drugs were dissolved in 5% Macrogol 400 solution; the Con and Mod groups were gavaged with a corresponding dose of 5% Macrogol 400 solution per day. The body weight and fast blood glucose (FBG) of all mice were measured weekly during the continuous administration for 8 weeks.

#### 2.8.2. Oral glucose tolerance test

The OGTT was conducted as described in a previous study (19). Briefly, the mice fasted for 8 h after 8 weeks of intervention, and all mice were orally administered with 2 g/kg glucose solution. The FBG of mice was measured at 0, 30, 60, 90, 120, and 180 min by using a blood glucose monitor (ACCU-CHEK Performa, India). The OGTT values were calculated based on the curve areas.

### 2.9. HPLC-QTOF-MS/MS analysis

The separation and identification of compounds were performed using an HPLC-QTOF-MS/MS system (20). Briefly, the samples were separated by a YMC-Triart C18 column (4.6 × 250 mm, 5 μm, Japan) at a flow rate of 0.8 ml/min. Formic acid water (A) and acetonitrile (B) were used as the mobile phase. The elution program was set as follows: 0 min, 5% B; 6 min, 9% B; 7 min, 18% B; 38 min, and 40% B. The elutes were interfered with a Hybrid Quadrupole-TOF Mass Spectrometer Triple TOF 5600+ system (SCIEX, USA) directly for mass identification. The MS and MS/MS data were acquired under the negative ion mode with an electrospray ionization resource. The full-scan mass spectrum was detected at a mass range of m/z 100–1,500 and 50–1,500 in the MS and MS/MS models.

### 2.10. Statistical analysis

Statistical analyses were carried out using the SPSS 22.0 software (IBM, Armonk, NY, USA). The plots were drawn with Origin 2019 (OriginLab, Northampton, MA, USA), and all data were expressed as mean ± SD (standard deviation). Significant difference among data was calculated by Tukey's-*b* and one-way analysis of variance (ANOVA). A *P*-value of < 0.05 was considered significant.

## 3. Results

### 3.1. Total phenolic content

The TPC of the crude extract and its fractions from RPA are listed in Table 1. The EAF showed the highest TPC, followed by the *n*BuF and DCF; the contents were 844.83, 210.11, and 106.22 mg GAE/g E, respectively, and the lowest TPC was found in the crude extract (26.70 mg GAE/g E). The results indicated that ethyl acetate had the best-enriching efficiency for the phenolics in RPA extract. Our previous research also found that ethyl acetate possessed better enriching efficiency for the polyphenols in *Acer palmatum* “Atropurpureum,” *Acer palmatum* Thunb (2), and *Ipomoea batatas* leaves (17) than *n*-butanol and chloroform. In addition, the TPC was much higher than that reported by Wang et al. (13) with a value of 58.60 μg/mg E, which could be explained by the difference in extraction solvents. Water is not a proper solvent to recover polyphenols due to their high polarity.

**Table 1:** Total phenolic content and the IC_50_ values for DPPH and ABTS^+^ scavenging ability and α-glucosidase inhibition of RPA extract and fractions.

**Samples**	**Total phenolics (mg GAE/g Fr.)**	**DPPH·(IC_50_, μg/ml)**	**ABTS^+^ (IC_50_, μg/ml)**	**α-glucosidase (μg/ml)**
Crude extracts	26.70 ± 0.78^d^	184.5 ± 0.71^c^	132.12 ± 10.35^b^	69.09 ± 2.04^b^
DCMF	106.22 ± 11.86^c^	420.80 ± 4.18^a^	173.17 ± 4.83^a^	21.35 ± 0.61^d^
EAF	844.83 ± 29.75^a^	19.57 ± 1.95^e^	7.82 ± 1.03^d^	7.27 ± 1.82^e^
nBuF	210.11 ± 6.45^b^	206.64 ± 3.34^b^	52.66 ± 1.11^c.^	29.63 ± 1.58^c^
Standards	–	27.35 ± 0.33^d^	10.04 ± 1.51^d^	197.01 ± 6.42^a^

Different letters (a–e) in the upper right corner of each column indicate significant differences between the data (*P* < 0.05).

### 3.2. *In vitro* free radical scavenging abilities

In *in vitro* antioxidant models, DPPH and ABTS^+^ scavenging abilities were used to assess the antioxidant activity of RPA fractions. As shown in Table 1, all fractions were able to scavenge DPPH and ABTS^+^. However, the EAF exhibited the strongest DPPH and ABTS^+^ scavenging abilities with IC_50_ values of 19.57 and 7.82 μg/ml, respectively. The DPPH scavenging ability was higher than that of the positive control quercetin, and the ABTS^+^ scavenging ability was comparable with quercetin, suggesting the excellent antioxidant potential of the EAF. The DPPH and ABTS^+^ scavenging abilities of *n*BuF and DCF were much lower than those of quercetin, especially for the DCF fraction, as the IC_50_ values were 420.80 and 173.17 μg/ml, respectively. Meanwhile, the DPPH scavenging abilities of DCF and *n*BuF were lower than that of the crude extract, while their ABTS^+^ scavenging ability was better than crude extracts. Considerable radical scavenging ability was also found by You et al. (21) by the 50% aqueous methanol extracts of raw and processed RPA. Therefore, the EAF was selected for further cell assays to evaluate oxidative injury protection.

### 3.3. Protection of EAF by H_2_O_2_-induced HepG2 cell oxidative injury

#### 3.3.1. Cell viability

**Figure 1:**
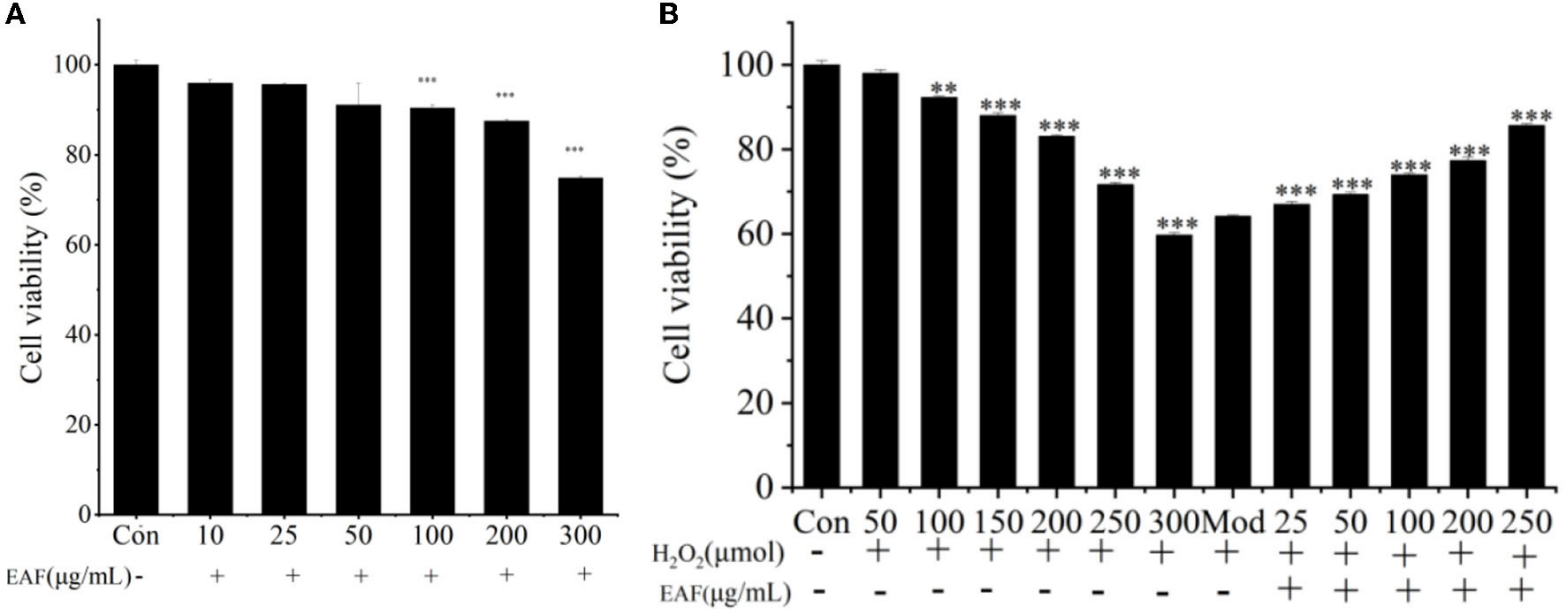
The cell viability of HepG2 cells in the presence or absence of the EAF **(A)**. The cell viabilzity of H_2_O_2_-induced HepG2 cells with or without the EAF **(B)**. The annotation ** and *** indicate the *P*-value of 0.05 and 0.01 compared to the Con group or the Mod group, respectively.

H_2_O_2_ is one of the commonly used substances for establishing oxidative damage in cells as it can penetrate cell membranes and cause cell damage and has been used in the osteoblasts, the nerve cells, the vascular endothelial cells, and the hepatocytes (9). Therefore, the H_2_O_2_-induced HepG2 cells oxidative damage model was used to further determine the oxidative protection effect of the EAF. The cytotoxicity of EAF toward natural HepG2 cells was presented in Figure 1A; with the increase in EAF incubation concentrations, the cell survival rate decreased gradually. Only 74.83% cell viability was achieved after treatment with 300 μg/ml of the EAF, and the viability at 200 μg/ml was 87.51%. Thus, 25–250 μg/ml was used for further experiments. The H_2_O_2_ damage degree described as cell viability is listed in Figure 1B, and dose-dependent cytotoxicity was observed. The cell viability was reduced from 97.99 to 59.71% after incubating with 50–300 μM of H_2_O_2_ for 4 h. Therefore, 300 μM of H_2_O_2_ was selected to damage HepG2 cells. To investigate the oxidative injury protection of the EAF, HepG2 cells were exposed to 25–250 μg/ml of the EAF before being stimulated with 300 μM of H_2_O_2_. It was clear that the EAF could significantly increase the cell viability (*P* > 0.05) and exhibit a dose-dependent relationship (Figure 1B). The cell viability reached up to 85.63% when 250 μg/ml of the EAF was added. The above results suggested that the EAF can protect HepG2 cells from the damage induced by H_2_O_2_.

#### 3.3.2. Effects on the levels of MDA, ROS, and antioxidant enzymes

**Figure 2:**
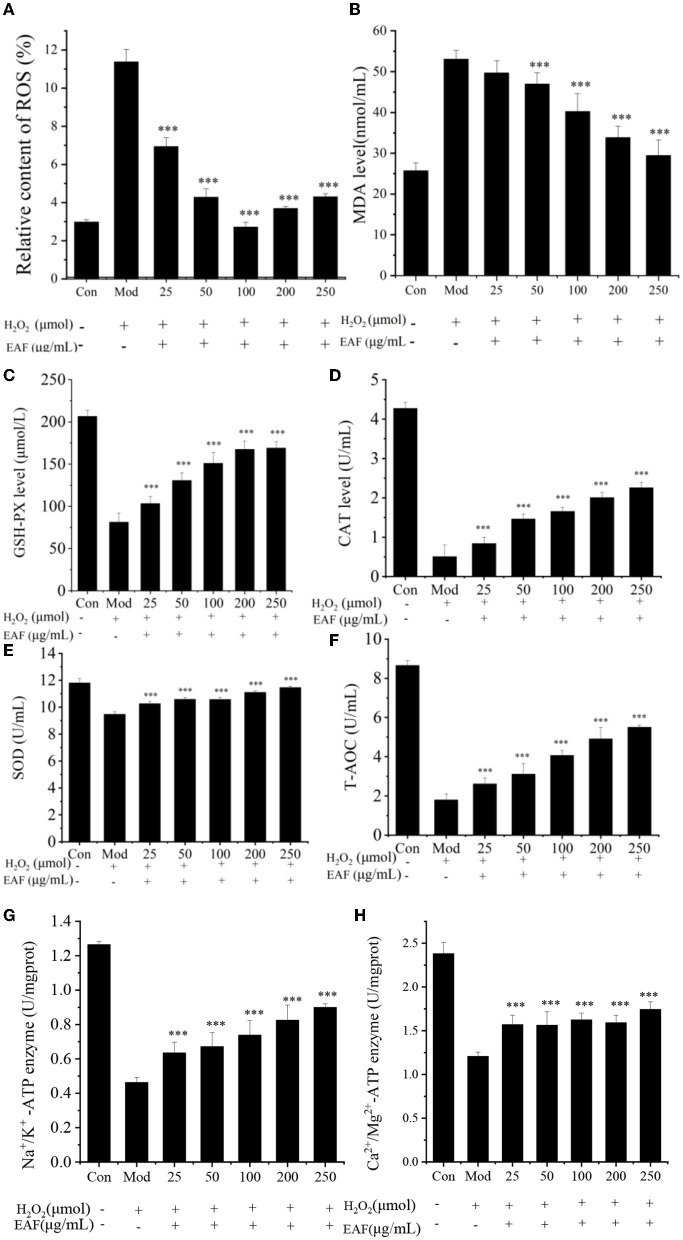
The MDA, ROS, CAT, SOD, GSH-Px, T-AOC, Na^+^/K^+^-ATP, and Ca^2+^/Mg^2+^-ATP levels in H_2_O_2_-induced HepG2 cells treated with different concentrations of the EAF **(A–H)**. Con: free of H_2_O_2_ treatment; Mod: induced by 300 μM H_2_O_2_; and EAF: induced by 300 μM H_2_O_2_ and different doses of the EAF. The annotation *** indicates a *P*-value of < 0.01 vs. the Mod group.

The MDA and ROS levels in liver cells are generally considered to be essential indicators of peroxidation and antioxidative defenses (14). Therefore, the effect of the EAF on the production of MDA and ROS in HepG2 cells was measured. As shown in Figures 2A, B, compared with the Con group, the H_2_O_2_ treatment significantly increased the ROS and MDA levels in HepG2 cells (*P* > 0.05), and they were clearly decreased when the cells were pretreated with the EAF. The ROS level was reduced from 11.37 to 2.72% when 100 μg/ml of the EAF was added. Meanwhile, the MDA level decreased from 53.03 to 29.44 nmol/ml when the concentration of the EAF was increased from 0 to 250 μg/ml. However, no obvious difference was observed between the lower dose (25 μg/ml) and the Mod dose EAF groups (*P* > 0.05). Therefore, the EAF could reduce the MDA and ROS levels and possess benefits for HepG2 cells against oxidative stress.

The GPX-Px, CAT, and SOD enzymes play a pivotal role in protecting cells from free radical damage, and the activation of the enzymes may help to scavenge active radicals and protect cells from high oxidative stress. The T-AOC reflects the total antioxidant levels of the enzymatic and non-enzymatic systems in the body, which are responsible for maintaining health (12). The effects of the EAF on the activities of GPX-Px, CAT, and SOD on H_2_O_2_-induced HepG2 cells are given in Figures 2C–E, and the activities of the antioxidant enzymes were remarkably reduced in the Mod group compared with the Con group. As expected, the EAF significantly increased the activities of GSH-Px, CAT, and SOD (*P* < 0.05). In particular, when the concentration of the EAF was 250 μg/ml, the expression levels of GPX-Px, CAT, and SOD were enhanced 2.07, 4.01, and 1.21 times, respectively (*P* < 0.05). The T-AOC was enhanced from 1.80 to 5.50 U/ml (illustrated in Figure 2F). Therefore, the EAF could regulate H_2_O_2_-induced oxidative stress by stimulating the activities of CAT, SOD, and GSH-Px and upregulating the T-AOC level. Ming and Dong (22) also found that intragastric administration of the RPA powder at doses of ~2–8 g/kg body weight/day could significantly reduce the serum MDA content and enhance SOD activity in Wistar rats.

#### 3.3.3. Effect on the activities of the energy metabolism enzymes

The ATPases of Na^+^/K^+^-ATP and Ca^2+^/Mg^2+^-ATP participate in the growth and reproduction of organisms, play an important role in signal transmission and energy metabolism, and even provide the necessary nutrients for cells (23). To further investigate the effect of the EAF on cell energy metabolism, the protective effects of the EAF on the Na^+^/K^+^-ATP and Ca^2+^/Mg^2+^-ATP enzymes were analyzed, and the results are shown in Figures 2G, H. The highest Na^+^/K^+^-ATP and Ca^2+^/Mg^2+^-ATP enzyme activities were found in the Con group with the values of 1.26 and 2.38 U/mg/protein, respectively. Induction with 300 μM of H_2_O_2_ could significantly reduce the activity of these two ATPases. Howver, the enzyme activity was individually increased by 1.44 and 1.94 times in the HepG2 cells pretreated with 250 μg/ml of the EAF prior to H_2_O_2_ damage. The enhanced Na^+^/K^+^-ATP and Ca^2+^/Mg^2+^-ATP enzyme activities were observed in oxidation-damaged HepG2 cells treated with *Capparis spinosa* L. (23). The results suggested that improved Na^+^/K^+^-ATP and Ca^2+^/Mg^2+^-ATP enzyme activity might contribute to the protection of EAF against H_2_O_2_ induced HepG2 cell oxidative stress.

#### 3.3.4. Inhibition rate of H_2_O_2_-induced HepG2 cell apoptosis

**Figure 3:**
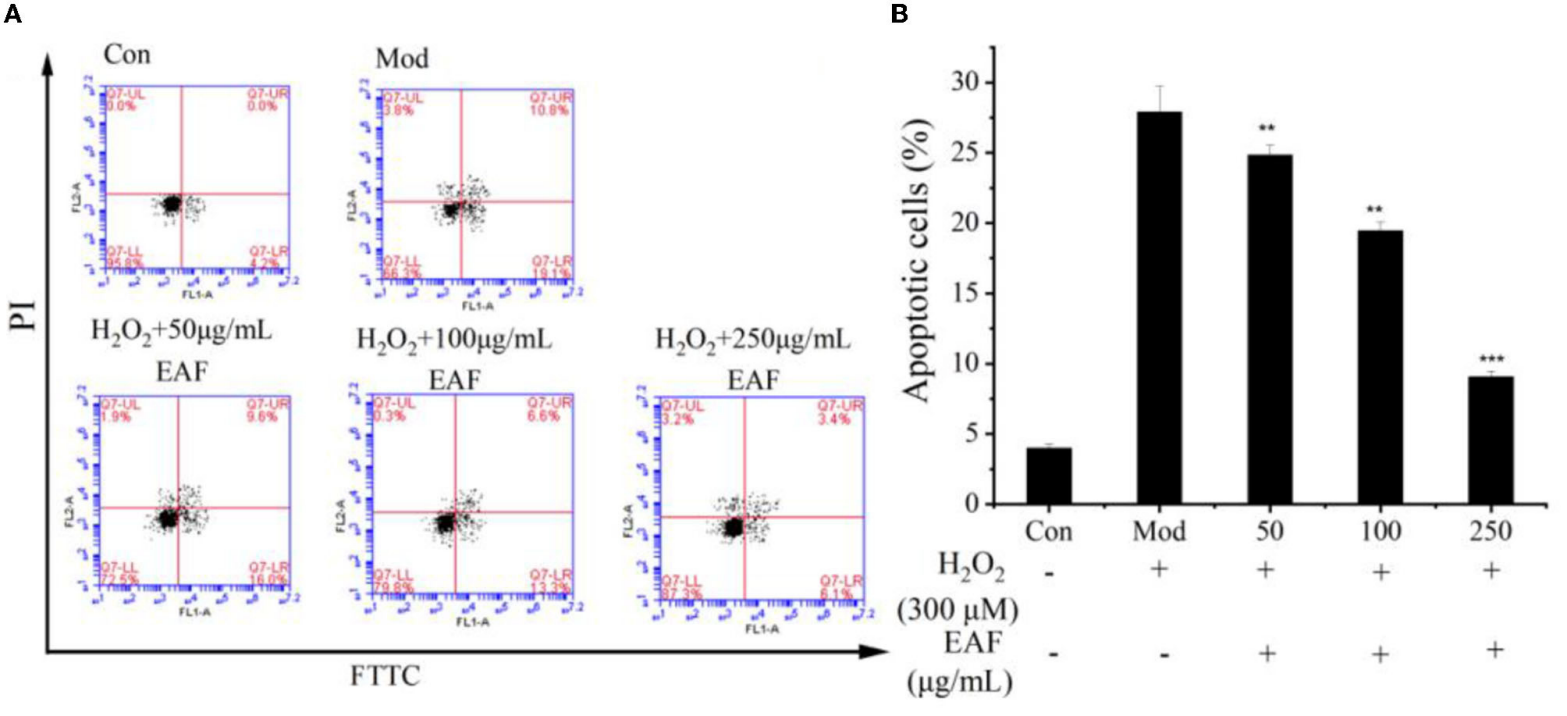
Annexin V-FITC/PI staining by flow cytometry. Representative image of fluorescence-activated cell sorting analysis **(A)**. Quantitative analysis of apoptotic HepG2 cells **(B)**. The annotation ** and *** indicate the *P*-value of 0.05 and 0.01 compared with the Con group or the Mod group, respectively.

Oxidative stress and cell apoptosis were known to have a close relationship, and the apoptosis of cells was seen as the end result of oxidative damage (14). From Figure 3, the apoptosis percentage detected in HepG2 cells cultured with H_2_O_2_ was 29.7%, which was much higher than that of natural HepG2 cells (Con group) (*P* < 0.05). Pretreatment with 50, 100, and 250 μg/ml of the EAF significantly attenuated the apoptosis percentage, and the values reduced to 24.8, 19.5, and 9.1%, respectively. The data indicated that the EAF could improve the apoptosis of H_2_O_2_-induced oxidative damage HepG2 cells and alleviate the oxidative stress of HepG2 cells.

### 3.4. α-glucosidase inhibition ability

α-glucosidase is an important carbohydrate hydrolase, and its inhibition activity can effectively retard the hydrolysis of carbohydrates and reduce the intestinal absorption of glucose. The α-glucosidase inhibitors are usually considered a promising approach to decrease fasting and postprandial blood glucose, and this approach was considered to prevent and treat diabetes (17). To investigate the potential capability of RPA extracts for diabetes treatment, the α-glucosidase inhibitory activity of RPA extracts was evaluated. As shown in Table 1, the RPA extract and its fractions (DCMF, EAF, and *n*BuF) were effective, and the detected IC_50_ values were 69.09, 21.35, 7.27, and 29.63 μg/ml, respectively. In addition, the EAF exhibited the highest α-glucosidase inhibitory when compared with other fractions and acarbose (IC_50_ = 197.01 μg/ml). The α-glucosidase inhibitory capabilities of DCMF, EAF, and *n*BuF were enhanced by 3.24, 9.50, and 2.33 times than that of crude extract, respectively. The α-glucosidase inhibition activity of RPA extracts and its fractions was highly correlated with the TPC and antioxidant activity (*R*^2^ = 0.76, 0.84), suggesting that phenolics and antioxidants in RPA contributed much to its α-glucosidase inhibition. Thus, based on these results, the EAF fraction was selected to further evaluate the effect on glucose uptake in IR-HepG2 cell models.

### 3.5. Promote glucose uptake in IR-HepG2 cells

**Figure 4:**
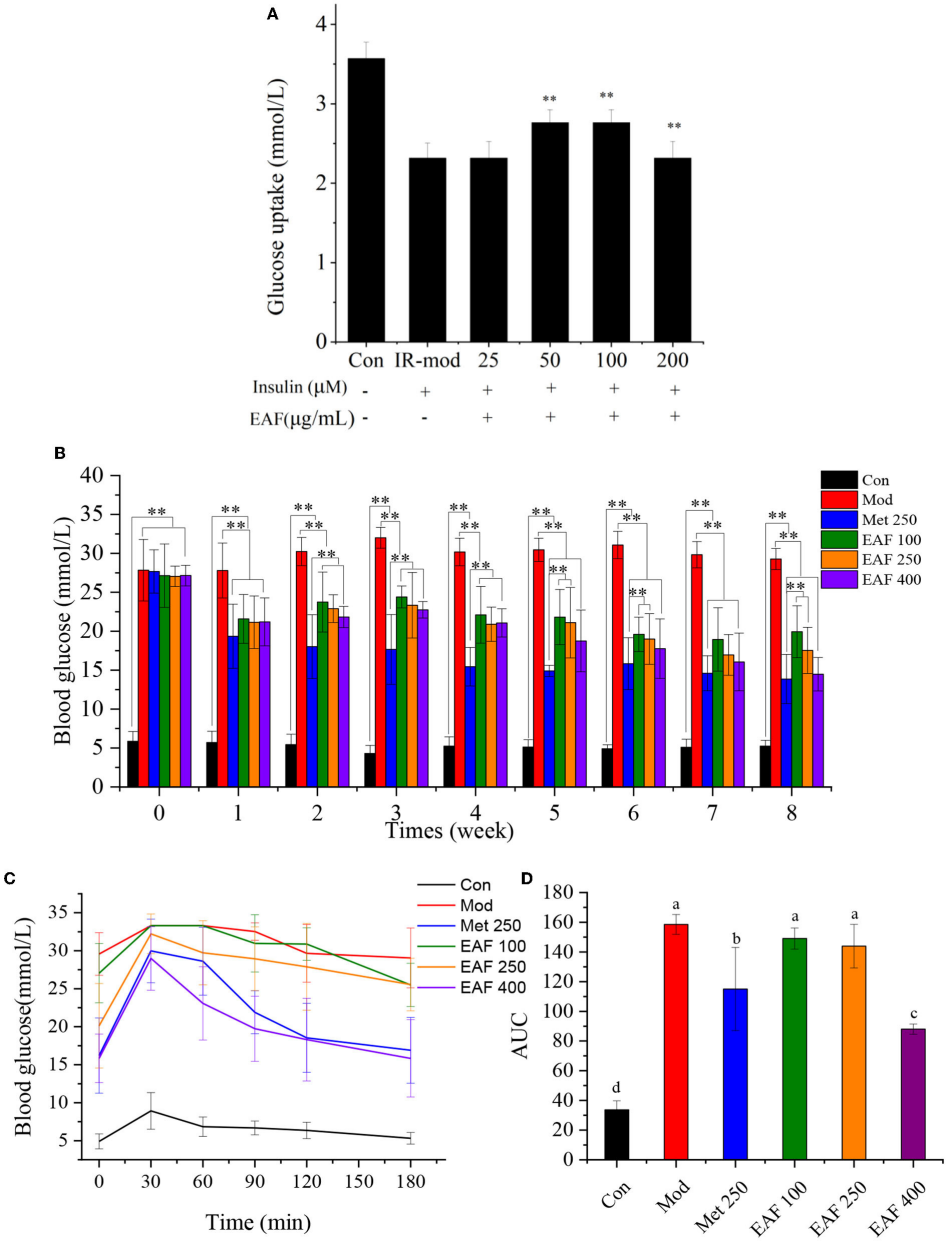
The effect of the EAF fraction on the glucose uptake of IR-HepG2 cells **(A)**, FBG **(B)**, OGTT **(C)**, and the area of AUC **(D)** in T2D mice. The different letters (a–d) in each column represent the significant difference (*P* < 0.05). **P < 0.05.

HepG2 cells are widely used in biochemical and nutritional studies as they can retain the morphology and functions in culture and still are suitable cell models to investigate IR (24). In this study, an IR-HepG2 model was used to estimate the ability of the EAF to modulate glucose uptake *in vitro*, and the results are shown in Figure 4A. After pretreatment with the EAF, the glucose uptake by IR-HepG2 cells was remarkably increased in comparison with the model group, which enhanced 0.78 mM when 100 μg/ml of the EAF was added (*P* < 0.05). However, the glucose uptake values of all other IR-HepG2 cell groups were lower than the Con group. Therefore, the EAF may treat T2D by increasing the glucose uptake in liver cells and hence the hypoglycemic effect *in vivo* is worth further evaluation.

### 3.6. Effects of the EAF on the FBG level and OGTT in T2D mice

As shown in Figure 4B, there were no significant differences in the initial FBG levels among the T2D mice in all groups. After oral administration for 8 weeks, the FBG level of the Mod group was significantly increased, while those of the Met and EAF groups were decreased and showed a dosage-dependent effect. In the last week, the FBG levels were reduced from 29.28 to 13.86, 19.98, 18.35, and 14.76 mmol/L in the Met 250, EAF100, 250, and 400 groups, respectively. The results suggested that the EAF pretreatment could improve the FBG levels of T2D mice.

An OGTT was often used to evaluate the abilities of the samples to regulate glucose metabolism *in vivo* (25). As given in Figures 4C, D, the blood glucose (BG) levels of all mice reached the peak at 30 min and then declined until 180 min, while the Mod group possessed clearly abnormal glucose tolerance. Compared with the Mod group, the AUC values of the Met 250, EAF100, EAF250, and EAF400 groups were reduced by ~27.46, 5.98, 9.18, and 44.46%, respectively. There was no significant difference between the EAF100 and EAF250 groups (*P* < 0.05). The results showed that supplementation of the EAF could enhance the glucose tolerance of the T2D mice, and the effect of the high dose of the EAF fraction (400 mg/kg body weight per day) was comparable with that of Met250.

### 3.7. Identification of phytochemical profiling

**Figure 5:**
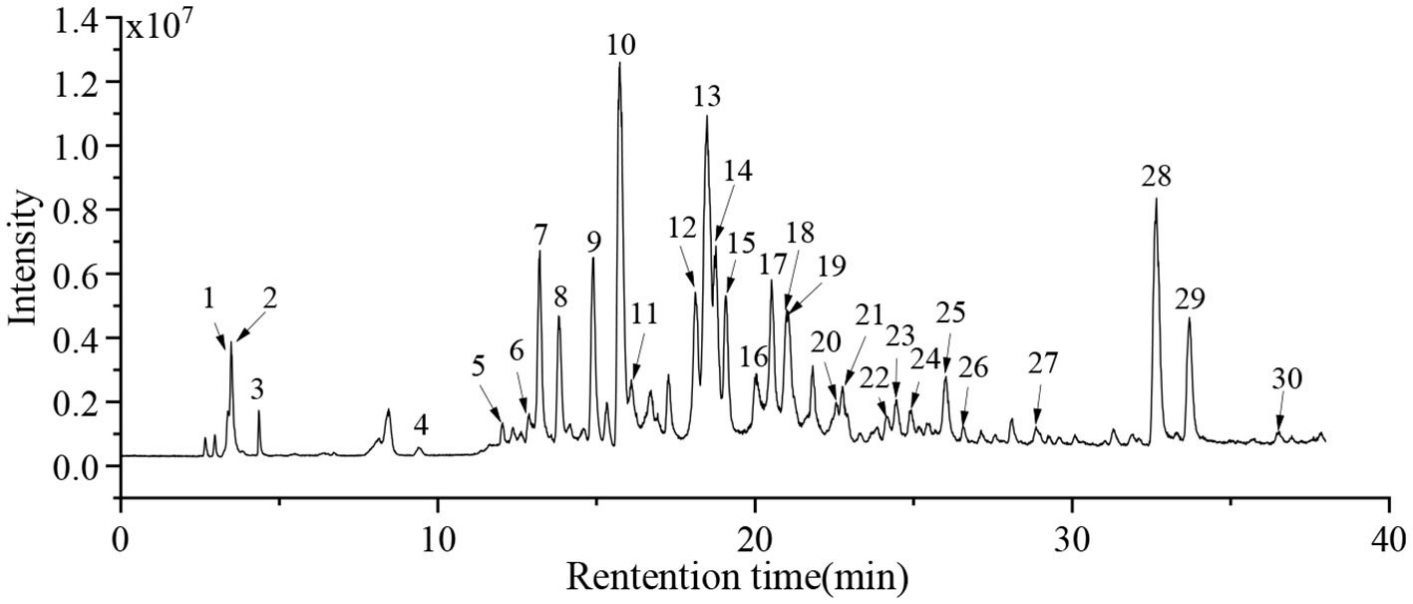
The total ion chromatogram (TIC) for the compounds in the EAF.

In this study, the HPLC-QTOF-MS/MS technology was performed to investigate the active compounds in the EAF. The compounds were identified by comparing the retention time, molecular formula, and MS/MS information with the references and database. The TIC spectrum of the EAF is displayed in Figure 5, and the MS/MS information is given in Table 2. A total of 23 compounds were identified in the EAF, including 5 flavonoids, 2 phenolic acids, 9 tannins, 3 terpenoids, and 4 other compounds.

**Table 2:** The chromatographic and mass data of detected compounds in EAF though HPLC-QTOF-MS/MS.

**No**.	**RT (min)**	**Formula**	**Found at m/z**	**Expected at m/z**	**Error (ppm)**	**MS^2^**	**Proposed compounds**
**Flavonoids**							
5	12.094	C_30_H_26_O_12_	577.1367	577.1352	2.6	451, 425, 407, 289, 161, 125	Procyanidin
7	13.189	C_15_H_14_O_6_	289.0738	289.0718	7.4	271, 245, 205, 179, 125	Catechin
13	18.442	C_15_H_18_O_17_	469.0430	469.0471	−8.9	393, 317, 241, 169, 125	Galloylmyricetin
21	22.763	C_24_H_30_O_12_	509.1684	509.1664	3.8	463, 341, 283, 197, 121, 77	Glochiflavanoside B
30	36.570	C_15_H_12_O_5_	271.0617	271.0612	1.8	253, 225, 197, 151, 125	Naringenin
**Phenolic acids**							
1	3.384	C_9_H_10_O_4_	181.0723	181.0718	3.2	163, 119, 110, 89, 71, 59	Dihydrocaffeic acid
4	8.409	C_7_H_6_O_5_	169.0151	169.0142	5.3	125, 97, 69	Gallic acid
**Tannins**							
6	12.854	C_14_H_10_O_9_	321.0260	321.0252	2.5	169, 125	Digallic acid
8	13.736	C_8_H_8_O_5_	183.0309	183.0299	5.2	168, 124, 78	Methyl gallate
11	16.113	C_34_H_27_O_22_	787.1034	787.0999	4.3	635, 617, 447, 295, 169	Tetragalloylglucose isomer
12	18.128	C_9_H_10_O_5_	197.0457	197.0456	0.8	172, 169, 124	Dimethyl gallic acid
14	18.775	C_30_H_32_O_15_	631.1684	631.1668	2.4	491, 399, 271, 169, 125	Galloylpaeoniflorin isomer
16	20.007	C_30_H_32_O_15_	631.1693	631.1668	3.9	509, 463, 313, 169, 151	Galloylpaeoniflorin isomer
17	20.530	C_30_H_32_O_15_	631.1679	631.1668	1.7	463, 313, 271, 169, 124	Galloylpaeoniflorin isomer
18	20.974	C_15_H_12_O_9_	335.0415	335.0409	2	183, 169, 124	Methyl digallate isomer
19	21.094	C_30_H_32_O_15_	631.1703	631.1668	5.5	313, 253, 169, 121	Galloylpaeoniflorin isomer
22	24.108	C_16_H_14_O_9_	349.0576	349.0565	3.1	349, 197, 169, 125	Dimethyl digallate isomer
24	24.848	C_37_H_36_O_19_	783.1818	783.1778	5.2	783, 631, 465, 313, 125	Digalloylpaeoniflorin isomer
25	26.011	C_16_H_14_O_9_	349.0578	349.0565	3.6	197, 169	Dimethyl digallate isomer
**Terpenoids**							
9	14.860	C_23_H_28_O_11_	479.157	479.1559	2.3	479, 357, 283, 121	Paeoniflorin isomer
10	15.726	C_23_H_28_O_11_	479.1564	479.1559	1	479, 449, 327, 283, 121	Paeoniflorin isomer
15	19.086	C_23_H_28_O_11_	479.1562	479.1559	0.7	479, 449, 431, 327, 165, 121	Paeoniflorin isomer
20	22.571	C_23_H_28_O_11_	479.1566	479.1559	1.4	479, 435, 357, 283, 121	Paeoniflorin isomer
27	28.873	C_27_H_38_O_13_	523.2263	523.2239	4	523, 493, 475, 375, 345, 327, 165	Mascaroside
28	32.660	C_31_H_34_O_14_	629.1906	629.1876	4.8	583, 535, 431, 265, 165, 121, 77	Mudanpioside B isomer
29	33.707	C_31_H_34_O_14_	629.1916	629.1876	6.4	583, 535, 431, 413, 265, 177, 165, 121	Mudanpioside B isomer
**Others**							
2	3.482	C_12_H_22_O_11_	341.1089	341.1089	0.1	179	Sucrose
3	4.365	C_6_H_8_O_7_	191.0207	191.0197	5.3	173, 129, 111, 87, 67	Critic acid
23	24.467	C_22_H_16_O_7_	391.0866	391.0823	10.8	229, 169, 124	6-(3,4-Dihydroxybenzyl)-5,7-dihydroxy-2-(4-hydroxyphenyl)-4H-1-benzopyran-4-one
26	26.546	C_21_H_34_O_10_	445.2095	445.2079	3.4	445, 293, 233, 149, 131	Pinen-10-Yl Vicianoside

#### 3.7.1. Flavonoids

Peak 5 was assigned as the procyanidin, and the MS/MS ion at 289 implied the existence of the epicatechin residue (20). Peak 7 with MS/MS ions at 271, 245, 205, 179, and 125 accounted for the characterization of catechin (26). Peak 13 with MS/MS ions at 169, 241, and 317 displayed the loss of a galloyl moiety and myricetin, which was proposed as galloylmyricetin (26). Peak 21 was suggested as glochiflavanoside B due to the same fragment ions in the literature (27). Peak 30 was identified as naringenin based on the fragment ions at 151 ([M–C_6_H_5_O–CO]^−^) (28).

#### 3.7.2. Phenolic acids

Peaks 1 and 4 were identified as dihydrocaffeic acid and gallic acid by comparing them with the standards.

#### 3.7.3. Tannins

Gallic acid derivatives and gallotannins contain one or more galloyl moieties and showed the characteristic fragment ions at 169.0133 ([gallic acid-H]^−^) and 125.0232 ([gallic acid-CO_2_-H]^−^) (20). Peak 6 with the MS/MS ion at 169 was suggested as digallic acid, which was generated by the dehydration condensation of two gallic acids. Peak 8 produced the fragment ion at 168 ([M–OH]^−^) and 124 ([M–CO_2_-OH]^−^) and was assigned as methyl gallate. Peak 12 was suggested as dimethyl gallic acid resulting from the MS/MS ions at 169 and 172 (26). Peak 11 was identified as tetragalloylglucose, with the MS/MS ions at 617 and 169 indicating the presence of 1 and 3 galloyl moieties (29). Peaks 14, 16, 17, and 19 showed the same MS ion and molecular formula and were tentatively identified as galloylpaeoniflorin and its isomers. The MS/MS ion at 313 implied the existence of galloyl glucose, which produced an ion at 169 by losing a glucosyl residue (29). The molecular weight of peak 18 was 152 Da ([gallic acid-OH–H]^−^), which is higher than peak 8 and was identified as methyl digallate. Similarly, peaks 22 and 25 were considered as isomers of dimethyl digallate, which were methyl-substituted compounds of peak 18 (2). Peaks 28 and 29 with MS/MS ions at 535, 431, and 121 were proposed as mudanpioside B isomers and have been found in the genus *Paeonia* (30).

#### 3.7.4. Terpenoids

Peaks 9, 10, 15, and 20 showed common MS ions at 479, which were identified as isomers of paeoniflorin (29). The MS/MS ions at 283 and 121 correspond to the loss of benzoic acid and glucose residues. Peak 27 was identified as mascaroside based on the MS/MS ions at 431, 375, 195, and 165 according to the literature (31).

#### 3.7.5. Others

Peak 2 was identified as sucrose using the standard. Peak 3 was proposed as citric acid, and the MS/MS ion at 129 indicated the loss of the carboxyl and hydroxyl groups (31). Peak 23 with MS/MS ion at 229 indicated the presence of one phenol and three hydroxyl groups and was suggested as 6-(3,4-dihydroxybenzyl)-5,7-dihydroxy-2-(4-hydroxyphenyl)-4H-1-benzopyran-4-one (32). Peak 26 was assigned as pinen-10-Yl vicianoside by matching MS/MS ions with the report by Nöst et al. (33).

## 4. Discussion

Many studies found that long-term drug therapies for patients with T2D would result in various side effects and generate drug resistance, while natural plant extracts have been demonstrated as alternative therapeutic agents or supplements to treat T2D by alleviating oxidative damage and hyperglycemia (34, 35). Currently, the exploration of novel antidiabetic drugs or dietary supplements from natural products is a hotspot in the research field of T2D. RPA, as a medicinal plant and has been reported to show various biological activities like antioxidant and hypoglycemic effects, but the main active compounds are still not clear. Therefore, the aim of this study was to investigate the phytochemical composition and the antioxidant and hypoglycemic activities of RPA *in vitro* and *in vivo*.

In this study, compared with other fractions, the EAF of RPA showed the highest total phenolic content, DPPH and ABTS^+^ scavenging capacities, and α-glucosidase inhibition ability, which were 31.6, 9.5, 16.9, and 9.5 times higher than the crude extracts, respectively. The Pearson correlation coefficients of the TPC with α-glucosidase inhibitory and DPPH and ABTS^+^ scavenging activities were −0.759, −0.753, and −0.816, which indicated that phenolics may be the main antioxidants and α-glucosidase inhibitors in RPA. A previous study also found that ethyl acetate displayed a stronger enrichment effect on phenolics in two *Acer palmatum* cultivars than *n-*butanol and water, which could be explained by the similar polarities (2). You et al. (21) reported that the DPPH scavenging capacity of 50% aqueous methanol extract from RPA was lower than that of our study with an IC_50_ value of 310 μg/ml. Simultaneously, HPLC-QTOF-MS/MS analysis suggested that flavonoids, tannins, and terpenoids were the main compounds in RPA, which was in accordance with the report of Xiong et al. (29). Galloylmyricetin, catechin, paeoniflorin, and gallic acid and its derivatives have been demonstrated to exhibit excellent inhibition abilities on free radicals and α-glucosidase and may have contributed to the biological activities of the EAF *in vitro* (3, 19, 24).

Furthermore, the effect of the EAF on oxidative damage was determined by an H_2_O_2_-induced HepG2 cell oxidative damage model. After pretreatment with the EAF, the levels of MDA and ROS in HepG2 cells remarkably decreased, and the activities of SOD, CAT, GPX-Px, T-AOC, Na^+^/K^+^-ATP, and Ca^2+^/Mg^2+^-ATP obviously increased. In addition, the addition of the EAF inhibited the apoptosis of HepG2 cells induced by H_2_O_2_. The results confirmed that the EAF could alleviate oxidative stress damage *in vitro*. Phenolics like tannins and terpenoids have been proven to prevent oxidative damage by improving antioxidant enzymes (36), which were abundant in the EAF. Moreover, flavonoids like procyanidin could improve energy metabolism by increasing ATP synthesis (37). As studied by Yuan et al. (9), paeoniflorin suppressed oxidative stress by enhancing the SOD and CAT levels in H_2_O_2_-induced HepG2 cells, and its derivatives were found in high contents in the EAF. Hydrolyzable tannins, as dominant ingredients in the EAF, have been reported to show a modulation effect on antioxidant enzyme levels and the activities of Na^+^/K^+^- and Mg2^+^-ATP in erythrocyte membranes (38). It has been found that catechin could improve the antioxidant enzyme (CAT and GSH-Px) contents *in vitro* and *in vivo* and protect HepG2 cells from apoptosis caused by H_2_O_2_ (39). Crispo et al. (10) also proved that methyl gallate reduced H_2_O_2_-induced apoptosis percentage in PC12 cells. In addition, oxidative stress was highly associated with insulin resistance (IR), which may influence glucose metabolism in cells. The result indicated that the EAF significantly enhanced the glucose uptake of IR-HepG2 cells. Similarly, pretreatment with procyanidin from grape seeds significantly increased glucose consumption in HepG2 cells from 36.78 to 55.92 μmol/mg cell protein (3). Many studies have also demonstrated that catechin and gallic acid and its derivatives treatment promoted glucose consumption in IR-HepG2 cells, and paeoniflorin treatment could alleviate IR in HepG2 cells (18). Therefore, the identified bioactive compounds in the EAF greatly contributed to improving H_2_O_2_-induced HepG2 cell oxidative injury and increasing glucose uptake in IR-HepG2 cells.

Db/db mice were used as a model to further investigate the hypoglycemic effect of the EAF *in vivo*. After consumption of the EAF for 8 weeks, the FBG level of db/db mice declined sharply by comparison with the Mod group and showed a dosage effect. Liu et al. (40) confirmed that the ethanolic extract from peony seeds led to a decrease in the glucose level in high-fat diet-induced T2D mice and suggested the same phenomenon in our research. The OGTT results exhibited that the AUC value of the EAF400 group dropped and was 44.46% lower than that of the Mod group, which revealed that the oral administration of the EAF could regulate insulin sensitivity and glucose metabolism of T2D mice, corresponding to our result *in vitro*. Flavonoids, phenolic acids, tannins, and terpenoids have been proven to show excellent effects in the management of T2D in various ways (5, 36). The phenolics and terpenoids from the EAF displayed good antioxidant and α-glucosidase inhibition activities, which might effectively reduce the absorption of glucose and improve IR and lower the FBG level in diabetic mice. As reported, the catechin-enriched extract treatment reversed the FBG level in db/db mice (19). Compared with the Mod group, the FBG level and the AUC value of 30 mg/kg paeoniflorin group decreased to 3.71 mmol/L and 10.19 after treatment for 4 weeks, and 4 paeoniflorin isomers were identified in the EAF. Based on the above results, it can be concluded that the EAF had a strong hypoglycemic effect *in vitro* and *in vivo*.

## 5. Conclusion

The EAF, as the best bioactive fraction from RPA extracts, could effectively scavenge free radicals, protect oxidative stress injury, enhance glucose uptake, and decrease hyperglycemia, which were attributed to the higher TPC (844.83 mgGAE/g) and components like flavonoids, phenolic acids, tannins, and terpenoids. The ability of the EAF to scavenge DPPH and ABTS^+^ and suppress α-glucosidase was higher than that of the corresponding standard (Vc and acarbose). The EAF treatment alleviated oxidative damage in HepG2 cells caused by H_2_O_2_ by increasing the SOD and CAT enzyme levels and reducing the production of MDA and ROS. In addition, the Na^+^/K^+^-ATP and Ca^2+^/Mg^2+^-ATP enzyme levels and cell apoptosis were also improved by the EAF. When the concentration reached 50 and 100 μg/ml, the EAF effectively increased the glucose uptake of IR-HepG2 cells. Here, a strong ability to decelerate FBG levels (from 29.98 to 14.76 mmol/L) in db/db mice was found at the gavage dose of the EAF 400 mg/kg/day. Therefore, the present study reveals that EAF has a great potential for development as a natural drug or a dietary supplement for the treatment of T2D.

## Data availability statement

The original contributions presented in the study are included in the article/supplementary material, further inquiries can be directed to the corresponding authors.

## Ethics statement

All animal procedures were approved by the Institutional Animal Care and Use Committee at Hunter Biotechnology, Inc. [Approval number: IACUC-2020-2574-01, Use license number: SYXK (zhe) 2022-0004]. The feeding and management were accredited by the Association for Assessment and Accreditation of Laboratory Animal Care (AAALAC) International (No. 001458).

## Author contributions

LZ: methodology, supervision, writing—review and editing, funding acquisition, and project administration. C-yP: investigation, validation, formal analysis, and writing. P-xW: methodology, investigation, and original draft. LX: software, investigation, and statistical analysis. J-hL: investigation and validation. XX: investigation and writing—review and editing. LL: investigation and methodology. Z-cT: conceptualization, supervision, and funding acquisition. All authors contributed to the article and approved the submitted version.
